# Near Real-time Natural Language Processing for the Extraction of Abdominal Aortic Aneurysm Diagnoses From Radiology Reports: Algorithm Development and Validation Study

**DOI:** 10.2196/40964

**Published:** 2023-02-24

**Authors:** Simon Gaviria-Valencia, Sean P Murphy, Vinod C Kaggal, Robert D McBane II, Thom W Rooke, Rajeev Chaudhry, Mateo Alzate-Aguirre, Adelaide M Arruda-Olson

**Affiliations:** 1 Divisions of Preventive Cardiology and Cardiovascular Ultrasound Department of Cardiovascular Medicine Mayo Clinic Rochester, MN United States; 2 Advanced Analytics Services Unit (Natural Language Processing) Department of Information Technology Mayo Clinic Rochester, MN United States; 3 Enterprise Technology Services (Natural Language Processing) Department of Information Technology Mayo Clinic Rochester, MN United States; 4 Gonda Vascular Center Department of Cardiovascular Medicine Mayo Clinic Rochester, MN United States; 5 Department of Internal Medicine Mayo Clinic Rochester, MN United States

**Keywords:** abdominal aortic aneurysm, algorithm, big data, electronic health record, medical records, natural language processing, radiology reports, radiology

## Abstract

**Background:**

Management of abdominal aortic aneurysms (AAAs) requires serial imaging surveillance to evaluate the aneurysm dimension. Natural language processing (NLP) has been previously developed to retrospectively identify patients with AAA from electronic health records (EHRs). However, there are no reported studies that use NLP to identify patients with AAA in near real-time from radiology reports.

**Objective:**

This study aims to develop and validate a rule-based NLP algorithm for near real-time automatic extraction of AAA diagnosis from radiology reports for case identification.

**Methods:**

The AAA-NLP algorithm was developed and deployed to an EHR big data infrastructure for near real-time processing of radiology reports from May 1, 2019, to September 2020. NLP extracted named entities for AAA case identification and classified subjects as cases and controls. The reference standard to assess algorithm performance was a manual review of processed radiology reports by trained physicians following standardized criteria. Reviewers were blinded to the diagnosis of each subject. The AAA-NLP algorithm was refined in 3 successive iterations. For each iteration, the AAA-NLP algorithm was modified based on performance compared to the reference standard.

**Results:**

A total of 360 reports were reviewed, of which 120 radiology reports were randomly selected for each iteration. At each iteration, the AAA-NLP algorithm performance improved. The algorithm identified AAA cases in near real-time with high positive predictive value (0.98), sensitivity (0.95), specificity (0.98), F1 score (0.97), and accuracy (0.97).

**Conclusions:**

Implementation of NLP for accurate identification of AAA cases from radiology reports with high performance in near real time is feasible. This NLP technique will support automated input for patient care and clinical decision support tools for the management of patients with AAA.

## Introduction

Worldwide prevalence rates of abdominal aortic aneurysms (AAAs) range from 1.6% to 3.3% for men older than 60 years [1]. Assessment of AAA may be performed by a variety of imaging tests, including ultrasound (US), computerized tomography (CT), and magnetic resonance imaging (MRI). In the United States, the prevalence of AAA has been reported as 2.8% among 9457 individuals screened by US [2]. Moreover, screening for early identification decreases the risk of aneurysm-related death and morbidity [1,3]. A prior study has shown that 4.5 ruptured AAA per 10,000 person-years were likely to have been prevented by screening, with an estimated 54 life-years gained per year of screening in a population of 23,000 men at risk [4].

The interpretation of imaging examinations is routinely reported in radiology reports as narrative text in electronic health records (EHRs) [5]. The automated extraction of information from narrative text can be accomplished by natural language processing (NLP) [6-8]. Prior studies have demonstrated high accuracy, sensitivity, specificity, and positive predictive value (PPV) of NLP for extraction of clinical concepts from narrative text in radiology reports [9-12]. Moreover, NLP is useful in cohort ascertainment for epidemiologic studies, query-based case retrieval, clinical decision support (CDS), quality assessment of radiologic practices, and diagnostic surveillance [5].

A previous retrospective cohort study from our institution developed a rule-based NLP algorithm for retrospective retrieval of AAA cases from radiology reports, which performed with high accuracy [12]. However, to the best of our knowledge, no prior study has demonstrated the use of NLP to identify AAA cases from radiology reports processed in near real-time. Hence, we tested the hypothesis that a rule-based NLP algorithm will extract AAA diagnosis from radiology reports in near real-time with high accuracy.

## Methods

### Study Settings

This study used Mayo Clinic radiology reports from May 1, 2019, to September 30, 2020.

### Study Design

**Figure 1:**
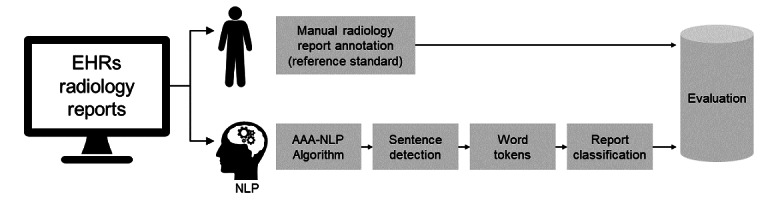
Study design. AAA: abdominal aortic aneurysm; EHR: electronic health record; NLP: natural language processing.

A rule-based AAA-NLP algorithm was developed for information extraction of AAA diagnosis automatically from radiology reports, including CT abdomen pelvis without intravenous (IV) contrast, CT chest abdomen pelvis angiogram with IV contrast, US abdomen complete, US aorta iliac arteries bilateral with doppler, MRI abdomen with and without IV contrast, and MRI pelvis with and without IV contrast. The rule-based NLP algorithm was developed using MedTagger and deployed in the institutional near real-time big data infrastructure to process relevant radiology reports. MedTagger is an open-source NLP tool that has been previously used in various clinical NLP applications [13]. MedTagger enables section identification, extraction of concepts, sentences, and word tokenization [14,15]. The AAA-NLP algorithm had 2 main components composed of text processing and report classification. AAA-relevant concepts were used to classify all reports (Figure 1).

A custom lexicon for AAA was identified by the study team through a manual review of radiology reports. Subsequently, this lexicon was mapped to corresponding concepts and their synonyms in the Unified Medical Language System Metathesaurus. The lexicon used for AAA identification included aorta abdominal aneurysm, aortic aneurysm abdominal, AAA, aneurysm abdominal aorta, and infrarenal aortic aneurysm. Each radiology report was then processed in near real-time by NLP. The AAA-NLP algorithm extracted both the lexicon and the contextual information of assertions, including negations or confirmations, from each radiology report. Textbox 1 displays the rules used by the NLP algorithm. The AAA-NLP algorithm classified subjects as AAA cases and controls without AAA.

To enable validation, the NLP output generated by near real-time processing of radiology reports was retrieved from the digital infrastructure by the information technology team and converted to a human-readable format for annotation. This annotation was performed by 2 trained physicians following written guidelines for standardization. The annotators were blinded to the diagnosis of each subject and to the results of the other annotator. In the written guidelines, AAA was defined as an aortic aneurysm diameter ≥3 cm by imaging as recommended by clinical practice guidelines [16].

The annotators reviewed the output from 120 processed radiology reports in 3 different training sets for iterative validation cycles to refine the algorithm. A total of 360 reports were reviewed. After abstracting and classifying the radiology reports, the information was entered and stored in a digital data set. Reports with a diagnosis of AAA were categorized as “case”; if there was no evidence of AAA or if an alternate diagnosis other than AAA was reported, the report was categorized as “control.” A board-certified cardiologist verified the information and resolved discrepancies in patient classification.

**Abdominal aortic aneurysm (AAA)–natural language processing rule and examples of text span.**

**Rule (any token + keyword for AAA + any token)**

Examples of confirmatory assertions

- Suprarenal *aortic abdominal aneurysm* which measures up to 5.2 cm
- Fusiform *infrarenal abdominal aortic aneurysm* terminating proximal to the aortobiiliac bifurcation, 56 mm, previously 56 mm
- There is a 5.7×5.1 cm *infrarenal aortic aneurysm* measured on image 175 of series 4

Examples of negated assertions

- *Negative* for *abdominal aortic aneurysm* or dissection
- *Abdominal aortic aneurysm* is *absent*
- *Negative* for thoracic or *abdominal aortic aneurysm*, dissection, penetrating atherosclerotic ulcer or intramural hematoma

### Statistical Analysis

The information extracted by the AAA-NLP algorithm from radiology reports in near real-time was compared to the reference standard manual review of radiology reports following written guidelines for standardization to calculate PPV, sensitivity, specificity, and F1 score. The formula to calculate F1 score was given as follows: 2 × ((PPV×sensitivity) / (PPV+sensitivity)) [5].

### Ethics Approval

This project was approved by the Mayo Clinic Institutional Review Board (approval number 21-006950).

## Results

Reports of 295 patients were validated in 3 different iterations. The data set for each iteration contained 120 reports, but 46 (16%) patients had more than one report. The reasons for more than one report for the same patient were imaging tests performed before and after repair procedures or surveillance for serial assessment of AAA (Table 1). There were no discrepancies regarding AAA diagnosis between 2 or more imaging reports from the same patient. Table 1 shows the distribution of demographic characteristics across AAA cases and controls. Cases and controls had similar ages in each of the iterative validation cycles, and most patients were Caucasian. AAA cases were more likely to have a history of smoking.

For evaluation of the AAA-NLP algorithm performance, 120 processed reports from each iteration were randomly selected. A total of 360 processed reports were reviewed by 2 physicians blinded to AAA diagnosis. There was 100% agreement for interactions 1 and 3. For interaction 2, the annotators disagreed on 1 report yielding a kappa coefficient of 92%. The disagreement was resolved by a board-certified cardiologist, creating the reference standard for comparison. The number of reports classified by the reference standard as true positives, false positives, true negatives, and false negatives in each iteration is shown in Table 2.

**Table 1:** Clinical characteristics and radiology report information.

Characteristic		Iteration 1			Iteration 2			Iteration 3	
		Case (n=31)	Control (n=52)	Case (n=44)		Control (n=59)	Case (n=59)		Control (n=50)
Age (years), mean (SD)		78.6 (11.1)	74.4 (12.4)	70.3 (8.4)		69.5 (14.1)	81.2 (8.5)		72.8 (10.4)
Male sex, n (%)		26 (84)	21 (40)	34 (77)		33 (56)	46 (78)		25 (50)
Caucasian, n (%)		31 (100)	52 (100)	42 (95)		54 (92)	58 (98)		48 (96)
**Comorbidities, n (%)**									
	Hypertension	24 (77)	39 (75)	31 (70)		27 (46)	46 (78)		37 (74)
	Hyperlipidemia	21 (68)	22 (42)	29 (66)		29 (49)	42 (71)		27 (54)
	Smoking history	29 (94)	24 (46)	35 (80)		23 (39)	50 (85)		25 (50)
	DM^a^	9 (29)	7 (13)	10 (23)		12 (20)	19 (32)		13 (26)
	PAD^b^	4 (13)	4 (8)	5 (11)		4 (7)	9 (15)		4 (8)
	CAD^c^	16 (52)	7 (13)	18 (41)		10 (17)	32 (54)		15 (30)
**Radiology reports**									
	Patients with ≥2 reports, n	18	7	13		1	3		4
	AAA^d^ diameter (cm), mean (SD)	4.6 (1.08)	N/A^e^	4.8 (1.3)		N/A	4.9 (1.2)		N/A
	Reports after AAA repair, n	2	N/A	9		N/A	8		N/A

^a^DM: diabetes mellitus.

^b^PAD: peripheral artery disease.

^c^CAD: coronary artery disease.

^d^AAA: abdominal aortic aneurysm.

^e^N/A: not applicable.

**Table 2:** Classification of abdominal aortic aneurysm from radiology reports during iterative validation.

	Iteration 1				Iteration 2				Iteration 3		
	Predicted case	Predicted control	Total	Predicted case		Predicted control	Total	Predicted case		Predicted control	Total
Actual case	TP^a^ 59	FN^b^ 6	65	TP 56		FN 2	58	TP 59		FN 3	62
Actual control	FP^c^ 1	TN^d^ 54	55	FP 4		TN 58	62	FP 1		TN 57	58
Total	60	60	120	60		60	120	60		60	120

^a^TP: true positive.

^b^FN: false negative.

^c^FP: false positive.

^d^TN: true negative.

**Figure 2:**
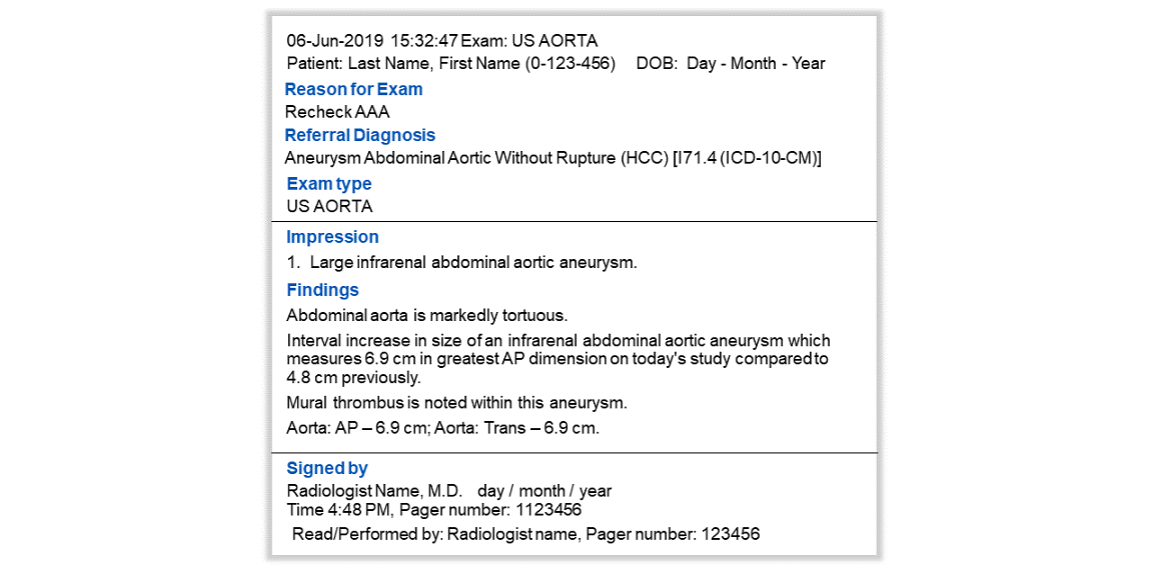
Example of deidentified radiology report with all sections. In this figure, section names are displayed in blue font. AAA: abdominal aortic aneurysm.

Radiology reports are composed of multiple sections. Figure 2 shows an example of a deidentified radiology report with all sections.

During the first iteration implementation, section ID number was used and section detection was challenging. For the second iteration, the algorithm was revised to include section header names for the filter criteria and solve sentence boundary issues. For the third iteration, section detection was implemented based on section names from our complete corpus using the frequency of normalized text with the tool lexical variant generation of the National Library of Medicine [17]. In a separate experiment, 203 additional radiology reports were reviewed by the annotators for evaluation of report section extraction, which resulted in accuracy of 0.96.

During this iterative refinement process, the report sections termed “reason for exam,” “referral diagnosis,” “exam type,” and “signed by” (Figure 2) were excluded, resulting in enhanced NLP algorithm performance. The report sections selected for processing were findings and impressions. During each iteration, the algorithm performance further improved. The performance metrics of the iterations are summarized in Table 3.

During the last iteration, 3 false negatives and 1 false positive contributed to the error analysis. False negatives were due to the complex nature of narrative text in these reports (ie, *no* significant interval changes in appearances of a partially thrombosed infrarenal AAA measuring 42×40 mm, extending to the level of aortic bifurcation and proximal common iliac arteries; *no* signs of rupture or impending rupture of the known infrarenal AAA; and *no* slightly increased size of fusiform infrarenal AAA). Additionally, the false positive was due to a typographical error, which was the report of a patient with an aorta diameter of 2.7 cm labeled as AAA, which does not meet the criteria for AAA (≥3.0 cm).

**Table 3:** Algorithm performance of each iteration.

Performance metric	Iteration 1 (n=120)	Iteration 2 (n=120)	Iteration 3 (n=120)
Sensitivity	0.91	0.97	0.95
PPV^a^	0.98	0.93	0.98
Specificity	0.98	0.94	0.98
F1 score	0.94	0.95	0.97
Accuracy	0.94	0.95	0.97

^a^PPV: positive predictive value.

## Discussion

### Overview

In this study, a novel rule-based NLP algorithm was developed for the extraction of AAA diagnosis from radiology reports and prospectively deployed in the institutional big data infrastructure for near real-time processing. Compared to the reference standard of manual review of radiology reports, the AAA-NLP algorithm extracted AAA diagnosis in near real time with high sensitivity, PPV, F1 score, specificity, and accuracy.

To the best of our knowledge, this study is the first to describe the use of NLP algorithms prospectively to extract AAA diagnosis in near real time from radiology reports. Clinicians, information technologists, and informaticians collaborated to refine the algorithm to improve performance. In previous studies, billing codes were used to find AAA cases [18,19]. However, in those studies, the cohorts were limited to patients with AAA who underwent procedures for aneurysm repair or had a history of ruptured AAA [18,19]. No prior studies using billing codes algorithms retrieved a broader spectrum of AAA diagnosis while also including patients presenting with uncomplicated AAA (ie, patients who did not undergo prior repair or who had not previously presented with ruptured AAA). In contrast, in this study, NLP automatically extracted AAA diagnosis from radiology reports prospectively and regardless of prior repair or rupture, thereby expanding the scope of computational approaches to include the detection of AAA cases prior to rupture or repair.

A radiology report consists of free text, organized into standard sections [5]. The American College of Radiology has published guidelines with recommendations for the use of sections for narrative (free text) entry in radiology reports [20]. NLP techniques enable the automatic extraction of information from narrative text [6-8]. Moreover, information extracted by NLP can be used to populate CDS systems automatically without the need for manual data entry and be better aligned with existing workflows such that radiologists can spend time interpreting images rather than filling out forms.

NLP is a computational methodology used for electronic phenotyping to extract meaningful clinical information from text fields [6,7,21]. In this study, we used NLP to process radiology text reports. The previous NLP algorithm used to find cases of AAA from radiology reports [12] was designed for retrospective cohort identification, whereas this report describes the prospective implementation of an NLP algorithm for input to a patient-specific CDS system for near real-time processing of radiology reports. Near real-time processing requires <3 milliseconds to process a document after a radiologist releases a report to the EHR [22]. The AAA-NLP implementation described in this study was developed within the existing digital infrastructure and can be used in clinical practice immediately without the need to retrain the algorithm. Additionally, the previously described algorithm [12] did not identify document sections in the radiology reports. By selecting specific sections for NLP information extraction, improvement in NLP performance was observed, as shown in the Results section. In the future, transformer-based NLP models [23,24] may be trained to interpret nuanced language, and ablation experiments [25] could be used to further evaluate these models.

The use of NLP algorithms has advantages compared to other methods. In comparison, the use of check box forms in radiology reports may require the development of new workflows [26,27]. The use of check box forms also requires the radiologist to direct attention away from the imaging interpretation process [26,27]. Manual entry of summaries of radiology findings in a check box can increase reporting time with decreased radiologist productivity [26,27]. Check box use could also result in the loss of important and clinically relevant descriptive information available only in the radiology narrative reports.

The rule-based AAA-NLP algorithm described in this study shows accurate detection of a broad spectrum of AAA cases prospectively in near real time from radiology reports, regardless of the presence of prior rupture or repair. This methodology will also potentially generate input for CDS to assist providers in managing patients with AAA by displaying the relevant information automatically at the point of care and in near real time for CDS tools. It will also support the automatic identification of cohorts for research purposes (eg, cohorts for clinical trials) and quality projects, and will support a learning health care system. NLP has been previously used for the identification of peripheral arterial disease and critical limb ischemia from narrative clinical notes of EHRs [21,28]. Therefore, it will also be possible to develop NLP algorithms for the identification of AAA cases from clinical notes in near real time.

In efforts to develop a learning health care system, Mayo Clinic has developed a robust big data–empowered clinical NLP infrastructure that enables near real-time NLP processing for the delivery of relevant information to the point of care via CDS [22]. Accordingly, we have deployed the AAA-NLP algorithm described herein to this digital infrastructure for translation to clinical practice. Importantly, the near real-time identification of patients with AAA by NLP responds to the American Heart Association scientific statement, which recommends the implementation of technologies to extract clinical information in real time that will promptly provide synopses of the information extracted [29].

### Limitations

This NLP algorithm was developed, tested, and implemented in a single tertiary medical center. Future studies should evaluate this algorithm at other institutions to demonstrate portability. A robust institutional digital infrastructure is required for the execution of near real-time processing of radiology reports [22]. Hence, the absence of adequate digital infrastructure may limit porting of this algorithm. For implementation, the analysis of radiology report architecture to enable the selection of document types and document sections may also be necessary for portability. Another potential challenge for porting this algorithm to other EHRs is differences in lexicons used for the extraction of the AAA concept across institutions. In mitigation, for this NLP algorithm, each lexicon was mapped to corresponding concepts and synonyms in the publicly available Unified Medical Language System Metathesaurus for standardization.

The algorithm was developed for the extraction of AAA diagnosis but not for the extraction of iliac artery or thoracic aortic aneurysms. Future studies should create and validate NLP algorithms for the extraction of thoracic and iliac artery aneurysms. The clinical criteria for AAA diagnosis involve a minimum diameter, but this NLP algorithm did not interpret the reported diameter. This is an area for future improvement in the algorithm, as clinical criteria for AAA may change over time. In this study, most patients were Caucasian. This was likely related to the ethnic distribution of communities in the Midwest, where this study was conducted [30,31]. Additionally, prior studies have reported a higher prevalence of AAA among Caucasians compared to other races [31,32]. There were differences in comorbidities of patients included in the 3 iterations. However, the NLP was developed for the extraction of the diagnosis of AAA and not developed for the extraction of associated patient comorbidities. The differences in patient comorbidities did not influence NLP performance for the extraction of AAA from radiology reports.

### Conclusions

Implementation of NLP for prospective identification of AAA cases from radiology reports in near real time with high performance is feasible. This near real-time NLP technique described will potentially be helpful for the generation of automated input for CDS tools to assist clinicians in the management of patients with AAA, quality improvement projects, and research (automated identification of cohorts).

## Abbreviations

AAA: abdominal aortic aneurysm
CDS: clinical decision support
CT: computerized tomography
EHR: electronic health record
IV: intravenous
MRI: magnetic resonance imaging
NLP: natural language processing
PPV: positive predictive value
US: ultrasound

## Data Availability

The data sets generated and analyzed during this study are not publicly available because participants in this study did not agree for their data to be shared publicly but are available from the corresponding author on reasonable request.
